# The Teeth as a Factor in Diagnosis

**Published:** 1889-10

**Authors:** Richard C. Newton

**Affiliations:** Mount Clair, New Jersey


### International Dental Journal.

Vol. X. October, 1889. No. 10.

### Original Communications.[1]

### THE TEETH AS A FACTOR IN DIAGNOSIS.[2]

BY RICHARD C. NEWTON, M.D., MOUNT CLAIR, NEW JERSEY.

At a recent meeting of the Practitioners' Society of New York, a paper was read on the care of the teeth from a medical practitioner's stand-point, and a discussion followed. The paper was printed in the *Medical Record*, and the accomplished editor of that journal considered the subject of sufficient importance to notice the paper and the discussion upon it.

In this editorial Dr. Schrady intimates that the importance of the surgical diseases of the teeth is not recognized by the medical profession, and in Dr. Sexton's paper it is said that the sooner our medical colleges insist upon a knowledge in their students of oral surgery before graduation the better for all concerned.

Now, here is a lamentable state of things, if it be true that surgeons generally are noticeably ignorant of oral surgery. Surgery, both special and general, has made great advances of late years, and no doubt oral surgeons are better informed than ever before

[1] The editor and publishers are not responsible for the views of authors of papers published in this department, nor for any claim to novelty, or otherwise, that may be made by them. No papers will be received for this department that have appeared in any other journal published in this country. The journal is issued promptly on the 15th of the month.

[2] Read before the New Jersey State Dental Society, at its nineteenth annual session at Asbury Park, July 18, 1889.

as to all surgical diseases of the teeth and jaws. It is certain that dental surgery is farther advanced than it has ever been, and that practitioners of dentistry are adding day by day to their knowledge and skill. I do not think that the medical aspects of the teeth are as well known as the surgical, nor that they have ever excited the general interest which their importance demands. It seems to me that we can spend a half-hour profitably in reviewing the literature of this subject, and in laying plans for future work towards elucidating and settling some of the questions that relate to the value of the shape, size, time of appearance, and other qualities of the teeth, in general diagnosis.

Can we tell anything about a person's constitution or habits, or anything relative to the presence of actual disease by merely examining the teeth? Not many years ago Mr. Jonathan Hutchinson published the results of his investigations in reference to the teeth in hereditary syphilis. Of course, you are all familiar with Hutchinson's teeth, which appear to be tolerably frequent in the syphilitic child. It is true that they are not always present in cases of undoubted syphilitic inheritance, and that teeth having these or similar peculiar malformations may be present in hereditary ataxia and rheumatism; and even sometimes in rickets.

Now, there is urgent need that the percentage of cases of hereditary syphilis showing Hutchinson's teeth should be determined. If possible, we should know wherein, if at all, the cases showing these teeth differ from those which do not show them. I might remark in passing that in such investigations the help of our dental confrères is absolutely necessary, or nearly so, before we can get at the truth. We want a great number of observations of people in health, or at least so well that they do not come to the physician. These important truths can be established only after years of study and observation; and I would fain implore you to record and report all your observations as to the peculiarities of the teeth. I remember some years ago a striking instance of the value of Hutchinson's teeth in determining the presence of a specific taint in a family of my acquaintance. Only one member of a family of four had these pronounced marks of the trail of the serpent, but undoubtedly the practised eye and skilful touch of a dentist might have detected certain peculiarities in the teeth of other members of this unfortunate quartette.

Dr. Harrison Allen and others in this country and several foreign writers have described so-called gouty teeth. Dr. Thompson, of Topeka, Kansas, read an address on this subject before the American Dental Association, in August, 1886, which was published in the *Dental Cosmos*, along with an excellent illustration of a cast showing the peculiarities of the teeth.

The different descriptions of the teeth, so far as I have read, agree, and there seems no doubt that when present, such teeth as Dr. Thompson described do indicate a gouty diathesis. Since gout is not a common disease in this country, it may not be out of place to remind you of its exceeding pathological importance. There is a form of Bright's disease which is known as gouty kidney. There are affections of the brain, eyes, liver, heart, and blood-vessels, not to mention the articulations, which are ascribed to the gouty diathesis. My own impression is that gout is not always recognized in this country, and that there is every probability that it will increase among us. Hence the great importance of any constant symptom which a patient may evince of the presence of a gouty tendency.

At the meeting of the Practitioners' Society referred to above, Dr. Gibney spoke of peg-shaped teeth, or peg teeth, and said that he often observed them in children with joint diseases. He thought that these teeth, instead of being called strumous or scrofulous teeth, would now be called tuberculous teeth. So far as this observation goes, it tends to show that there is a peculiar shape to the teeth in tubercular or scrofulous children, although I understand that the teeth of consumptives are as a rule good, at least in the earlier stages of the disease. This fact might be used in favor of the present theory to the effect that phthisis is at first a local and not a constitutional disease.

In cretins the teeth come very slowly, the process often occupying many years, and being accompanied frequently by an offensive salivation and convulsions.

In rickets the first dentition is delayed until after the child is twelve months old, whereas in hereditary syphilis the first dentition occurs before the sixth month. Hence the time of the eruption of the teeth enables us to distinguish between two diseases that were formerly confounded,—often to the great injury of the helpless patient.

In Bright's disease the teeth are said to be abnormally sensitive. This is a very important point, on account of the great frequency and fatality of this complaint, and on account of the difficulty which we often encounter in making a diagnosis early, the opportune time. It is true that examinations of urine are, as now made, very exact. But we have the authority of Professor H. C. Wood for saying that in some cases of morbus Brightii the urine is unaltered, at least for considerable periods.

In scurvy and in lead poisoning the appearance of the teeth and gums is pathognomonic.

In idiots the teeth present every anomaly of appearance and disposition. They grow and are shed without any regard to order or rule. In "Seguin on Idiocy" a case is mentioned of an idiot girl of eighteen who was shedding her fifth set of teeth.

In idiots the number of teeth also seems to be frequently either above or below the normal. Writers tell of double and even triple rows of teeth in the jaws of these unfortunates. The pitted, notched, and stained teeth which we often see are said to indicate that their owner suffered from fever or other exhausting disease during the calcification of these teeth. A curious difference in the health and vigor of the teeth in the same jaw has often been noted, some teeth being soft and friable, and others firm and vigorous. This condition can generally be traced to a fit of sickness which seized the patient during the calcification of those teeth found to be unhealthy.

To sum up this review of the scanty literature of the subject, we may say that (to quote a writer in the *Dental Cosmos*) this is a subject of which we know a little and suspect much. The little we know is of undoubted value to the general practitioner; but thousands of observations are yet needed. It is no wonder that those who have looked into this matter deplore the apathy of the profession in general with regard to this important subject. People do not as yet sufficiently value their teeth, nor see to it that their children grow up with good teeth. It seems odd that such a distinguished writer and teacher as Professor N. S. Davis should have said at the International Medical Congress, in September, 1887, that he had said over twenty years before that the teeth are as important as any other part of the organism, and that dentistry would come to be a recognized specialty of medicine. I say it seems odd, because his saying it in the manner and at the time that he did would seem to show that there is still considerable doubt, even in the medical mind, about the importance of the teeth; and also some doubt about the dignified position which dentistry should occupy.

Now, if the teeth are as important as the eyes, why is it, to take a particular instance, that we have volumes of literature upon albuminuric retinitis, and nothing upon the teeth in Bright's disease? If the experience of others coincides with that of my friend, Dr. Watkins, who says that he has observed cases of Bright's disease in which the teeth cannot retain a filling because they are so exquisitely sensitive, it is high time that medical men knew something more definite of this phenomenon. Dr. Sexton says that in fifteen hundred cases of ear disease he found that perhaps one-third owed their origin or continuance in a greater or less degree to diseases of the teeth. One would infer that the aurist, at least, should be well up in dental pathology and therapeutics. We sadly need more literature on the teeth, and who can supply it better than some of the learned and scientific men who have done so much for American dentistry?

Another peculiarity of the teeth and jaws has excited some comment, and led to considerable research during the last few years. I refer to the V-shaped and saddle-shaped jaws. What interest as practitioners of medicine have we in these anomalies? Do these misshapen jaws and protruding teeth evince any special phases of constitution? Do they come from preventable causes, and do they affect the general health? I think that it has been pretty well settled that they do not belong to any especial diathesis. They seem to come from several causes, and to affect the health mainly because they are apt to cause mouth-breathing, a pernicious and hurtful habit. I have said that they cause mouth-breathing; I should probably have said perpetuate mouth-breathing;—for breathing through the mouth seems to be one of the principal causes of the condition. The enlarged tonsils which are so common in our American children, by more or less occluding the posterior nares, engender the habit of breathing through the mouth. The mouth is almost constantly open, especially during sleep. The lower lip hangs down and draws the extremities of the upper lip against the cuspids and bicuspid teeth. This constant pressure, especially during the eruptive period, forces the teeth inward, makes the upper jaw narrower, and pushes the incisor teeth forward and upward. This deformity can generally be cured if taken in time.

The narrow jaws of Americans have excited much discussion, without, so far as I know, any satisfactory explanations having been brought forward. We can say that our sharp physiognomies are a race peculiarity; but this is not an explanation. Probably, when we have learned to live better and eat more wholesome food, these peculiarities will measurably disappear. We exercise our jaws too little in chewing our food, whatever other exercise they may get. It is said that the custom of chewing gum is beneficial; that it develops and preserves the teeth and widens the jaws.

As to irregular and crowded teeth in general, it is acknowledged that they decay and perish sooner than even and regular teeth. This must be partly due to the mechanical difficulty in keeping them clean; but also in part, I fancy, to the fact that they are in themselves more perishable. In my opinion the two principal reasons for the irregular and perishable teeth of our children are (1) sending them to school too early, and (2) feeding them improperly. As to the latter cause, so much has been said and written that I need not take up your time to enlarge upon it. To take a familiar example, let it suffice to say that the children must have phosphates. Baron Liebig says that one thousand pounds of wheat contains twenty-one pounds of phosphates, and one thousand pounds of white flour contains five-and-a-half pounds of phosphates. In other words, by our absurdly wasteful process of preparing flour, we have removed about three-fourths of the bone, tooth, nerve, and brain food from our children's bread.

I will fortify my second assertion by quoting Dr. Kingsley, who says, "The primary cause, so far as the individual is concerned, of any general disturbance in the development of the permanent teeth showing itself, particularly in their malposition, is directly traceable to a lesion of or enervation of the trigeminus nerve; that it is an interference, more or less prolonged, with one of the prominent functions of that nerve, and operating at its origin. The function of the trigeminus, thus stimulated or interrupted, is that which supports, regulates, and governs the nutrition of the tissues to which its terminal branches are distributed."

Professor Austin said, in one of his lectures on the fifth nerve, "The nervous centre in which the trigeminus is implanted is of all nervous centres the one which, in the human subject, is most liable to congenital imperfection of the kind which necessitates a break-down in its governing functions at a special crisis in the development of the organism."

From the *Boston Medical and Surgical Journal* of June 3, 1886, I clip the following: "Among the hard-worked pupils of the Paris public schools the teeth become deteriorated in a few weeks after entry. The second dentition is often premature," etc. Dr. J. L. Williams has shown the evil effects of any mental strain upon the teeth, both in increased sensibility and more rapid decay. Dr. D. M. Parker says that these same changes are always apparent in men who are training for athletic contests. Mr. Hutchinson, in his lectures on the pedigree of disease, says that, "In the discussion on rickets which took place in the Pathological Society of London two years ago, several speakers drew attention to the fact that this disease is very rare in sunny climates, although infant inanition from poor food may be common." We may certainly believe it probable that our climate and the narrow, sunless streets of our large towns take their share in the production of what was once known on the Continent as the English disease." In other words, the mal-assimilation caused by impure air, confinement, and want of sunlight, as well as poor regimen, produces a disease, one of whose most pronounced characteristics is interference with dentition.

The American school system too often turns out a fairly proficient student, whose education is indeed an expensive if not a useless luxury, since his health has been seriously impaired. The integrity of his most precious organs, including his teeth, have been sacrificed. He has already an assortment of neuralgias, dyspepsias, lumbagos, and headaches,—why? In great measure because his education was forced and his health neglected years ago. Day says, in his admirable work on headaches, "I think that the period of the second dentition is the earliest period when instruction requiring brain-work can be safely pushed. The period of the second dentition must be reckoned the most important period of childhood, from a developmental point of view. They (the people who would force education) indicate a grievous lack of capacity to comprehend the fragility of a structure which is incapable of bearing any additional burden."

By the English factory laws the development of the teeth is taken as the best standard of physical capability, and as a more reliable test of age than height, because it is well known that the tallest children are generally the most weak and fragile.

Gentlemen, this is too important a subject properly to be set forth in the time allotted to me. However, I cannot for a moment doubt that much of the nervousness of Americans depends upon faulty digestion, and that this primarily depends upon poor teeth and want of exercise and fresh air.

A recent writer, high in authority, says that the loss of the teeth is a frequent cause in the aged of rheumatoid arthritis of the tempero-maxillary articulation. The inference is of course that false teeth, by preserving the shape of the jaw and preventing muscular atrophy, would obviate the tendency to this complaint, the consequences of which are very inconvenient and often serious.

It is possible that conservatism is now being carried somewhat too far in dentistry as well as in general surgery. Nevertheless, our greatly increased knowledge of fermentative micro-organisms has given us a correspondingly increased range of choice. As surgeons we do not fear, as our predecessors did, that our patient's life may be sacrificed in trying to save a limb,—because we can tell much more closely his chances of recovery with or without an operation. In like manner you can often choose whether a tooth shall be preserved that would in former years certainly have been sacrificed. Before dentistry had reached its present high level, bad teeth were not in such inexcusably bad form as they are at present.

Admitting the deterioration of mankind physically, it is known that he is longer-lived now than ever. A great part of the credit for this must certainly be given to the dentist. We must in fairness acknowledge that dentistry has conferred upon mankind benefits whose magnitude is beyond our powers of comprehension.

As one of your writers has said, a true view of a dentist's duty to his patient makes that duty begin and end with life itself.

